# Single neuron responses in NCL, MVL, and Wulst during the observation of videos of conspecifics support population feature coding

**DOI:** 10.3389/fnbeh.2026.1736261

**Published:** 2026-02-26

**Authors:** Sara Santos Silva, Daniela Bühn, Paxton Hall, William Clark, Jonas Rose, Michael Colombo

**Affiliations:** 1 Neural Basis of Learning, Faculty of Psychology, Institute of Cognitive Neuroscience, Ruhr University Bochum, Bochum, Germany; 2 Department of Psychology, University of Otago, Dunedin, New Zealand; 3 Department of Neurobiology, Harvard Medical School, Boston, MA, United States

**Keywords:** conspecific recognition, dynamic stimuli, feature-based coding, multimodal integration, pigeon, sparse coding, tectofugal pathway, thalamofugal pathway

## Abstract

Social visual processing in vertebrates employs sophisticated neural mechanisms ranging from categorical face cells to distributed sparse coding systems. In primates, recent evidence supports a “tuning landscape” model where neurons signal distances to prototypes in high-dimensional space rather than functioning as simple category detectors. However, social visual processing in non-mammalian animals remains poorly understood. We recorded single-unit activity from three functionally distinct pigeon brain regions—mesopallium ventrolaterale (MVL), visual Wulst, and nidopallium caudolaterale (NCL)—while birds viewed dynamic videos of conspecifics and control shapes performing courtship, eating, flying, and walking behaviors. Despite finding visually responsive neurons in all regions, we observed no categorical distinction between conspecific and control stimuli. Instead, population analyses revealed discrete temporal modulations corresponding to specific motion features—bowing, wing-flapping, head-bobbing—suggesting feature-based rather than categorical encoding of visual information. Sound-modulated visual units were significantly more prevalent in MVL than Wulst, indicating earlier multimodal integration in the tectofugal pathway than previously recognized. The absence of differential responses in NCL during passive viewing, contrasting with clear modulation in visual areas, suggests that this region is less involved in the automatic analysis of visual features. These findings suggest that avian visual structures use sparse coding principles that are similar to the visual cortex, where populations encode specific features through coordinated but brief neural responses rather than sustained categorical signals.

## Introduction

Conspecific recognition represents a fundamental challenge in visual neuroscience, requiring rapid discrimination of socially relevant stimuli in the environment. Across vertebrate taxa, this capacity supports the acquisition of spatiotemporal information that is a prerequisite for the animal to understand and respond to changes in its surroundings (Kiyokawa et al., 2022; Greer et al., 2025).

The neural mechanisms underlying social recognition have been extensively studied in primates, which has revealed that the processing strategies used by neurons extend beyond simple categorical coding of visual stimuli. Early investigations of the primate inferior temporal cortex identified face-selective neurons (Perrett et al., 1982; Desimone et al., 1984), organized in discrete patches (Tsao et al., 2006), which lead to influential face cell models of social visual processing. However, the application of information theory has consistently demonstrated that sensory neurons in the higher visual cortex employ distributed sparse coding mechanisms rather than simple categorical responses, supporting monkeys’ exceptional capabilities in object discrimination (Rolls and Tovee, 1995).

Recent theoretical advances have unified sparse coding principles within a broader “tuning landscape” framework (Ponce et al., 2019; Wang and Ponce, 2022). Rather than functioning as categorical detectors, visual sensory neurons signal distances to “prototypes”—specific combinations of visual features within high-dimensional representational space. The model bridges visual encoding mechanisms with hippocampal cognitive mapping theories, where place cells encode locations in multidimensional task-relevant spaces (Aronov et al., 2017). The framework predicts that responses that appear to be categorical, a priori, actually emerge from the population-level dynamics in feature space rather than from individual neurons encoding abstract categories of visual stimuli.

Dynamic social stimuli are used to reveal additional mechanisms in population coding beyond those that are detectable using presentation of static images. McMahon et al. (2015) demonstrated that during naturalistic movie viewing, macaque face patch neurons exhibit a remarkable diversity of selectivity, with spatially neighboring cells responding selectively to distinct aspects of social scenes. These included facial identity, proximity relationships, and body movements. Individual neurons maintained consistent responses across repeated presentations while encoding different dynamic features of social interactions, suggesting that natural social processing employs population codes that are more sophisticated than those apparent during the static presentation of images.

The avian visual system provides a compelling comparative framework for understanding the neural coding principles that are used to interpret conspecific behavior across vertebrate evolution. Pigeons possess sophisticated visual capabilities that support complex social behaviors such as social learning, individual recognition and the formation of social hierarchies (Nagy et al., 2013; Bouchard et al., 2007; Delacoux et al., 2025). Behavioral investigations demonstrate successful conspecific categorisation from photographs and videos, including discrimination between familiar and unfamiliar individuals (Shimizu, 1998; Nakamura et al., 2003; Ware et al., 2015; Wilkinson et al., 2010).

Birds have two major visual pathways like those found in mammals (Karten, 1969; Shimizu and Bowers, 1999). The tectofugal pathway is functionally analogous to the mammalian colliculo-pulvinar-cortical pathway (Li et al., 2007; Frost, 2010). Previous studies using static stimuli in operant categorization tasks revealed that a population of neurons in the mesopallium ventrolaterale (MVL), a higher center of the tectofugal pathway, supports the decoding of different categories of visual stimuli with diverse features (Azizi et al., 2019; Clark et al., 2022a,b). The visual Wulst in the thalamofugal pathway is comparable with the striate visual cortex (Bischof et al., 2016) and is believed to mainly process simple features such as orientation in pigeons’ lateral surroundings (Ng et al., 2010).

In social interactions, the richness of sensory cues at a pigeon’s disposal is much higher than what static stimuli can provide. There is a possibility that the pigeon’s ability to identify a conspecific relies on a wide array of visual features, but also on other sensory cues, such as sound. We recorded single-unit neural responses in pigeons, from three different regions: MVL, along the tectofugal pathway, the visual Wulst along the thalamofugal pathway and the nidopallium caudolaterale (NCL), the equivalent structure to the mammalian prefrontal cortex (Güntürkün, 2005; Güntürkün and Bugnyar, 2016; Nieder, 2017), while head-fixed and body-restrained birds watched videos of conspecifics performing four different behaviors: courtship, eating, flying, walking. The videos were corrected for low-level features to ensure that any selectivity was not due to differences in luminance or spatial frequency. By using dynamic stimuli with natural sound accompaniment, we observed that the population coding of dynamic social information during passive fixation is most consistent with a sparse code for visual feature information across the recorded visual brain regions.

## Materials and methods

### Subjects

The study used eleven pigeons (*Columba livia)* of undetermined sex, as subjects. The birds were housed in individual cages, in a colony room maintained at 20°C, with a light-dark cycle of 12 h. During data collection, the pigeons were kept at their free-feeding weight, having unrestricted access to a mixture of grit, wheat, sunflower seeds, peas and corn. Water intake was controlled such that access to water was removed for 12 h prior to an experimental session.

All experimental, animal handling and housing procedures were carried out in accordance with the University of Otago’s Code of Ethical Conduct for the Manipulation of Animals and approved by the Animal Ethics Committee of the same entity.

### Experimental apparatus

**FIGURE 1:**
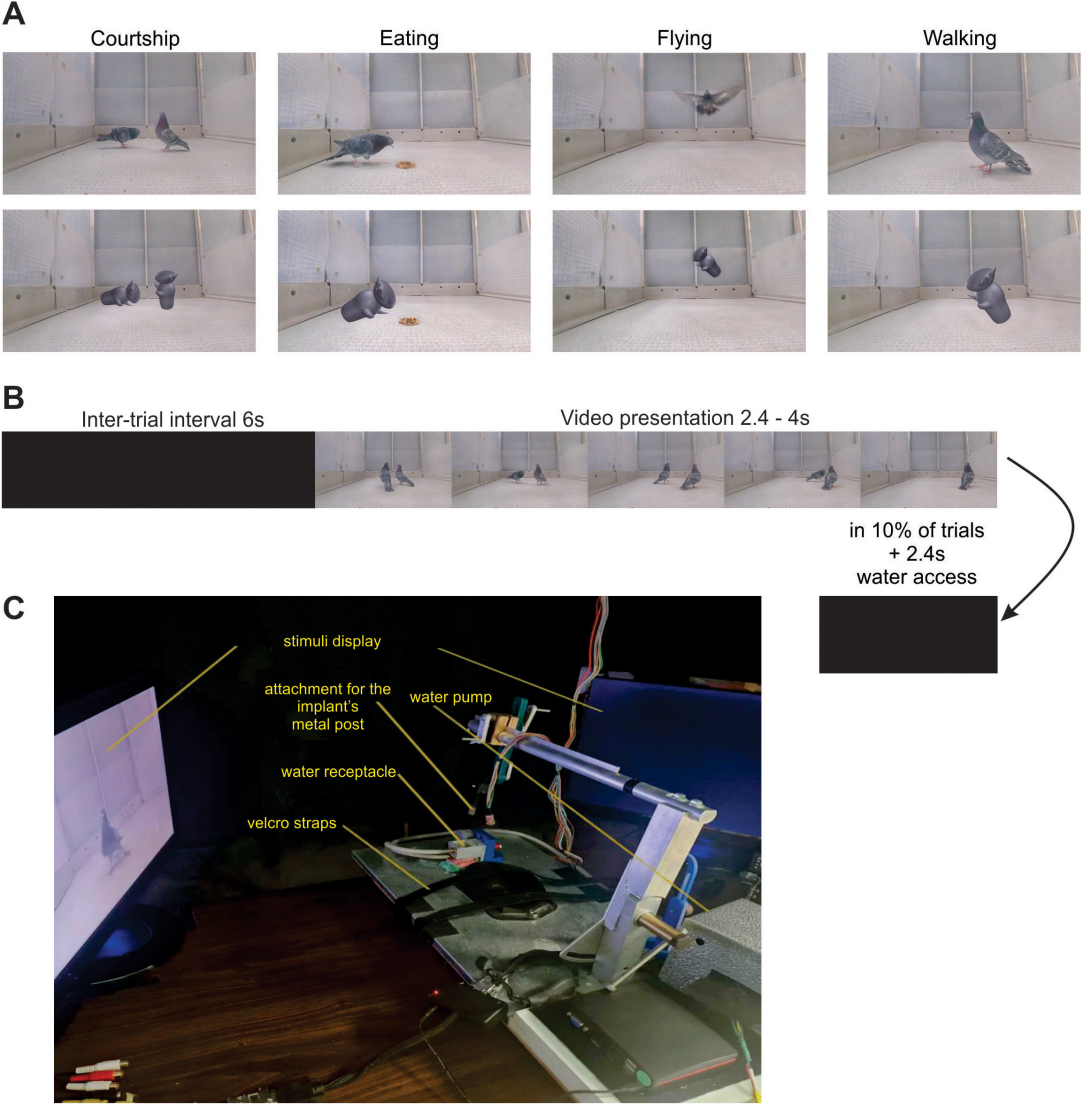
Experimental setup and visual stimulation. **(A)** Example frames from four videos of the experimental stimulus set. Each frame on the top row is part of a video that depicts one of the target behaviors: courtship, eating, flying and walking. The bottom row depicts the corresponding frame in the version of the video where the action is performed by the greeble. The greeble videos preserve the motion performed by the pigeon. **(B)** Schematic representation of the stimulation protocol. A trial consisted in the presentation of one video. In 10% of the trials of a given session, after the video presentation, the pigeons had 2.4 seconds of access to water. Subsequently, there was a 6 second inter-trial interval. During the inter-trial interval and the period of water access, the screens remained black. **(C)** Labeled photograph of the experimental setup.

The experiment was performed with head-restrained and body-restrained pigeons. A custom-made metal frame was built to accommodate the fixation of the animal and the administration of water. The apparatus contained a water receptacle that, connected to a pump, filled and emptied automatically (Figure 1C). The pump was controlled using custom code, written in MATLAB, jointly with the OTBR (Otto and Rose, 2023) and Psychophysics toolboxes (Brainard, 1997).

There were also two monitors for stimuli display: they were laterally placed at 74 cm, on either side of the pigeons’ eyes, with an azimuth that centered the images on the optic axis (Nalbach et al., 1990) of the pigeon. The monitors were gamma corrected.

### Task/visual stimulation

Head-restrained and body-restrained pigeons passively viewed a prepared set of videos that displayed pigeons, or a control shape, performing the following behaviors: courtship, eating, flying and walking. The sequence of videos was pseudorandomized, guaranteeing that the same behavioral category wasn’t displayed consecutively more than three times. Each video was anteceded by a six second inter-trial interval, and was shown six times per session, three times on the left monitor and three times on the right monitor. In total, there were 432 trials, and a session took approximately 75 min to complete. A random 10% of the trials were followed by 2.4 s of access to water (Figure 1B).

### Stimuli

The stimulus set of this experiment is composed of videos, recorded in the lab (GoPro Hero 4 session), with durations ranging from 2.2 to 4 s, depicting four different behaviors: courtship, eating, flying and walking (Figure 1). Within each category, four different videos were created to exemplify the same behavior, such that there is some variation in the way it is represented. Although all videos were recorded in the same aviary enclosure, which gave them a very similar background and illumination, they were recorded so that the same action was performed in different places on screen. Occasionally, different camera angles were used.

For each video, we created a corresponding control video in which the background was preserved but the motion that characterizes the targeted behavior was performed by a 2D shape, called a greeble (Gauthier et al., 1998). The greebles were edited to be the same size as the pigeons and were given the dominant color of a background subtracted frame of one of the pigeon videos. That is, the color of the greeble was the average color of the pigeon. All control videos were edited using Adobe After Effects (Adobe Inc., 2023).

Additionally, the stimulus set included four auxiliary videos, showing two pigeons engaging in no specific behavior. The *two-pigeon stimulus set* was included to disambiguate whether neuronal responses to courtship behavior represented courtship behavior or the presence of two animals on screen.

Furthermore, each video had a version where the natural noise, produced with the recorded behavior, was preserved, and a version in which the video was silent. Hence the developed stimulus set consisted of 72 videos, out of which 36 were visually unique.

### Processing of stimuli

**FIGURE 2:**
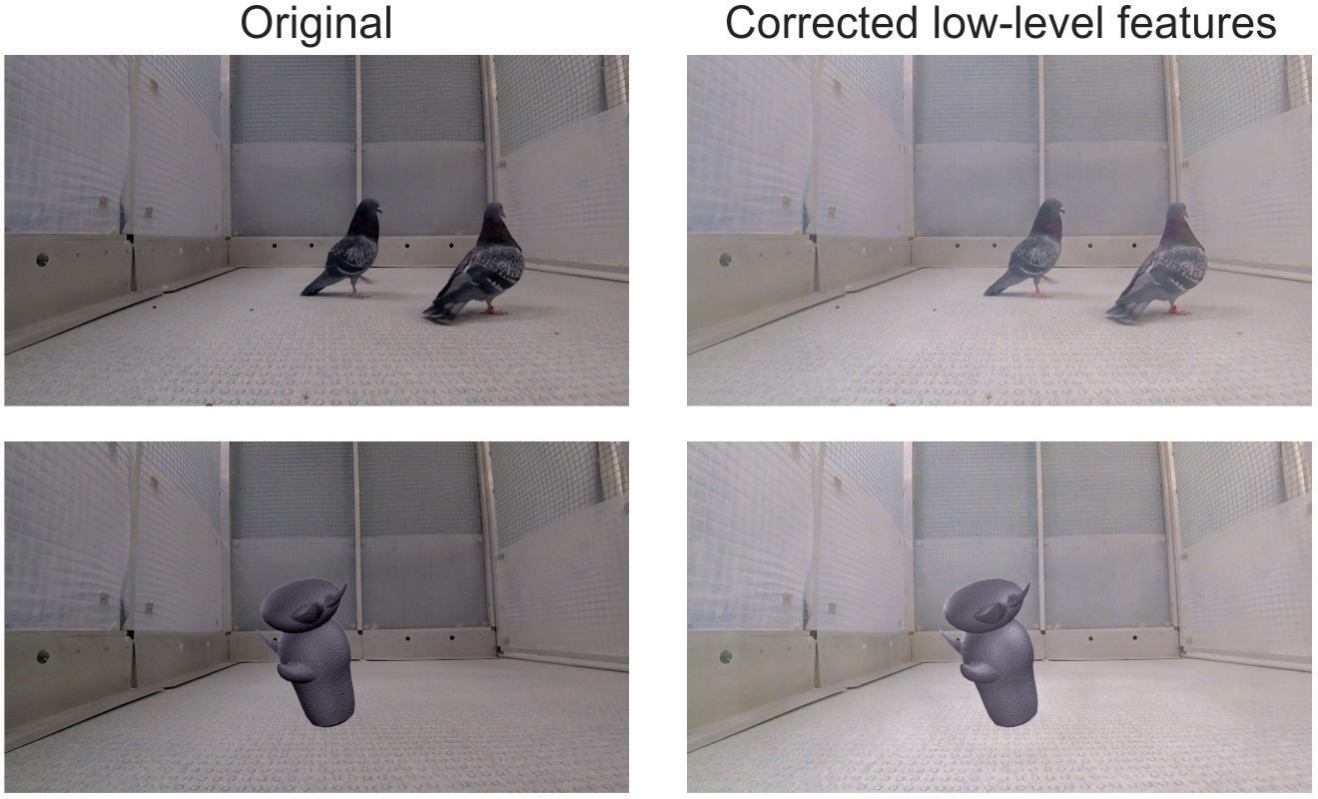
Example video frames before and after low-level feature correction. Images on the left column are original frames from a courtship video (top left), and a greeble walking video (bottom left). The images on the right column are the corresponding frames after the equalization of luminance and spatial frequency.

An important aspect to consider when comparing the neural responses to stimuli ascribed to different categories is whether the observed differences are driven by low-level stimuli features, such as luminance and spatial frequency (Clark et al., 2022a,b). Therefore, we averaged the luminance values across all frames of all pigeon videos. Equalization was performed separately for the background and the subject. Subsequently, the average values were applied to the control (greeble) videos. The spatial frequency was equalized across all videos (pigeons and controls) but without changing the individual orientation (angle of the light and dark bars, obtained from the fast Fourier transform of the image) of each frame (Figure 2; Willenbockel et al., 2010). We chose this type of equalization to preserve the frame’s natural look and minimize any visual aberrations when they were parsed together to compile the video. Each frame was converted to the HSV color space and the low-level feature transformations were applied to the value layer (Dal Ben, 2023). The correction of luminance and spatial frequency were done using MATLAB (Mathworks Inc., Natick, MA United States) and the SHINE toolbox (Willenbockel et al., 2010, with some code adapted from the SHINE_color toolbox; Dal Ben, 2023). All videos were captured and displayed at 30 frames per second (a subset of the stimuli is provided in Supplementary material).

Lastly, we quantified how visually dissimilar each video was from its control, on a frame-by-frame basis. This way, we created an expectation of which moments of the video could drive an accentuated difference in neuronal responding, purely by the difference the pixel space occupied by the pigeon and the greeble on the image (further details and results in Supplementary material: frame-by-frame correlation).

### Surgery

We performed stereotactic surgery to implant one microdrive, with a probe of eight 25 μm formvar-coated nichrome wires, per animal. For NCL [anteroposterior (AP), 5.5; mediolateral (ML), (±) 7.5], two pigeons (K4, X27) were implanted on the left hemisphere and one (K6) on the right hemisphere. For MVL [AP 10.5; ML (±) 6], two pigeons (K8, JIM) were implanted on the left hemisphere and one (PAM) on the right and. For the visual Wulst [AP 11; ML (±) 3], two pigeons (C1, O4) were implanted on the left hemisphere and three (O9, O8, X15) on right hemisphere. The coordinates for implantation followed histological and inactivation studies (Karten and Hodos, 1967; Kröner and Güntürkün, 1999).

The birds were anesthetized with a mixture of Ketamine (30 mg/kg) and Xylazine (6 mg/kg), administered intramuscularly. A topical anesthetic (10% Xylocaine) was applied to the scalp and an incision was made to expose the skull. Subsequently, a small craniotomy was made over the target structure, and the dura matter was removed. For each bird, a microdrive was installed over one of the target regions: NCL, MVL or Wulst. The tips of the wire bundles were lowered until the beginning of the target region during surgery. To support the implant, stainless steel screws were inserted into the skull and one of them also served as a ground screw. The microdrive was secured to the skull and to the screws with dental acrylic (Jet Acrylic, Lang Dental). Around the microdrive, we added a short metal post, that was used to attach the animal to the frame of the experimental apparatus. In the days following the surgery, the animals were given analgesics and allowed to recover. On the first 3 days of recovery, for pain control, the animals received an intramuscular injection of Carprieve (4 mg/kg) and the wound margin was sprayed with 10% Xylocaine.

### Neuronal recordings

We used probes of eight wires to record extracellular single-neuron activity. Before each recording session, the probe was checked for neuronal signals: pairs of two wires were tested to assess which combination of recording wire and local reference yielded the best signal isolation. If the single-to-noise ratio of the isolated unit was at least 2:1, the unit was considered for recording. The subject was not submitted to any task related visual stimulation, during this process.

Sampling at 20,000 Hz, the signal was amplified using a Grass P511K amplifier (Grass Instruments, Quincy, MA, United States) and a 5 Hz notch filter was applied. The signal was digitized using a CED (Cambridge Electronic Design, Cambridge, United Kingdom) interface. Jointly with Spike2 software, spike sorting was done using the system’s template matching capabilities.

The experimental paradigm ran on a separate computer that sent triggers of relevant events to the CED system, which allowed the alignment of the visualization paradigm with the neural data.

After each recording session, the probe was advanced 42 μm and the animal was returned to its home cage. In case it wasn’t possible to isolate a unit that satisfied the inclusion criteria, the probe was advanced 21 μm and the session didn’t take place.

### Statistics and reproducibility

In this experiment, we analyzed single-cell activity in three regions of the pigeon brain (Wulst, MVL, NCL), during the passive observation of videos, depicting pigeons performing different behaviors and their respective controls. We were interested in quantifying neuronal responses, of each one of the regions, to video features shared amongst multiple stimuli. For instance, we wanted to assess neural modulation congruent with the presentation of a video where a specific behavior is performed by a pigeon. It is possible to argue that the features we expect to drive neuronal responses are nested within each video. Considering the nested structure of the stimuli and the repeated measurements of the same neuron, we used several generalized linear mixed models (GLMMs) to assess selectivity, at the single-neuron and population level. When interpreting the models, statistical significance was considered at a level of α = 0.05.

For all neurons, only the trials where the videos were shown on the screen contralateral to the pigeon’s implanted hemisphere were considered for analysis, due to the almost complete decussation of the optic nerve in this species and its impact in the ascending visual projections (Güntürkün and Hahmann, 1999). Therefore, for a given neuron, we recorded its activity to the same visual stimulus six times: three presentations of the video with sound and three without sound. Data analysis was performed in R (R Core Team, 2024) [packages: glmmTMB, DHARMa, (Hartig, 2024) emmeans (Lenth, 2025)] and MATLAB (The MathWorks Inc., 2024).

### Neural data analysis

For each unit, spike counts were binned in 200 ms bins. To determine how the data of a recorded region was distributed, we fitted two null models: one with the poison distribution and the log link function, and another with the negative binomial distribution and log link function (Salinas Ruíz et al., 2023).


nullModel=spikeCounts∼1+(1|trialNr)+(1|neuronID)

The model fitted with the negative binomial distribution had a better fit to the data than the Poisson model (see Results). Subsequently, we used the DHARMa package in R for residual diagnostics and zero-inflation testing. The test results informed the decision to fit all the models used in the study with the negative binomial distribution and the log link function (R package glmmTMB).

#### Single neuron analysis

At the single-neuron level, we tested if firing rate modulations, relative to baseline, could be attributed to the main effect of “actor” (pigeon or greeble), “behavioral category” (courtship, eating, flying or walking) or their interaction. We also measured the contribution of the auditory component of the videos by including an interaction term between “actor” and “sound” (sound On or Off). The random effects component of the model had the trial number as a random intercept: it was included to account for the possible visual adaptation to the stimuli, throughout the session.


m1=spikeCounts∼actor*behavioralCategory+actor*

s⁢o⁢u⁢n⁢d⁢(1|t⁢r⁢i⁢a⁢l⁢N⁢u⁢m⁢b⁢e⁢r)

A model was fitted to the binned spike counts of each neuron. The dataset consisted of the middle two-seconds of the baseline and the entire video presentation of every trial. The baseline of the trials of the first courtship video was set as the intercept of the *m1* model.

The output of the model was used for hypothesis testing: the *p*-value for each parameter was calculated with the Wald Z-statistic. Furthermore, comparisons between model estimates were done through estimated marginal means (R package emmeans) and the resulting *p*-values were adjusted with the Bonferroni-Holm method. Neurons that had a singular fit were excluded from analysis.

From the output of the model, a unit was classified as *actor-selective* if the comparison of firing rate between the baseline and video presentation resulted in a significant interaction effect between, exclusively, one actor and two or more behaviors or, only, in a significant main effect for one of the actors; the classification of *behavior-selective* was given to units that responded significantly to a specific behavior, for both pigeons and greebles; lastly, *actor-and-behavior-selective neurons* were the ones that only presented a significant interaction between one behavior and one actor. Furthermore, it was possible to assess whether the presence of sound contributed to the observed firing rate modulations. We counted how many neurons fitted this classification and compared their proportions between the three regions: the comparisons were performed using Chi-Squared tests, with Holm–Bonferroni correction, or the Fisher’s Exact test, with Holm–Bonferroni correction, if the expected counts in a group were below 5.

#### Population analysis

In the set of neurons that were tuned to stimuli, we were interested in accessing which moments of the video drove the observed neuronal modulation. For this analysis, we fitted a model that explored the influence of the actor and the moment in the video on firing rate.


m2=spikeCounts∼actor*BinNrInTrial+(1|

trialNumber:BinNrInTrial)+(1|subjectID:neuronID)

We ran the model *m2* on the subset of units labeled actor selective, tuned to the actor pigeon, for every region. As in the previous test, the neural activity recorded in each trial was segmented in bins of 200 ms. We tested the same neuronal subset for each of the 16 videos (four videos for each of the behaviors) and detected where the activity of the subpopulation was significantly different from baseline. Then, multiple comparisons were performed, to assess if the predicted spike counts for the temporal intervals of the pigeon videos, in which neural activity was significantly modulated from baseline, differed between pigeons and greebles. As in the previous test, the neural activity recorded in each trial was segmented in bins of 200 ms.

It is relevant to emphasize that the random effects component of the model *m2* contains a random effect for neuron identity, which accounts for differences in baseline firing for each unit. Neuron identity is nested within subjects, which accounts for subject-specific effects and prevents us from drawing conclusions based only on one animal (Yu et al., 2022).

To further assess how the dynamics of the recorded population change, through the progression of each video, a population state-space analysis was conducted per region. Conceptually, a population state-space analysis starts by representing the activity of a single neuron, at every recorded moment, as a point on a plane. Every neuron generates its own plane, which maps the population activity onto a *n*-dimensional space, where *n* corresponds to the total number of recorded neurons. If there is information encoded at the population level, the spiking activity of several neurons will covary according to a smaller set of variables than the number of recorded units. Using a principal components analysis, dimensionality reduction was performed. The first three dimensions were preserved and their projection on the data was used to define trajectories for each condition (16 videos × 2 actors). The trajectories characterize the instantaneous populational firing rate, through the progression of each video.

The population state-space analysis was performed on all task-engaged neurons (MVL *n* = 49; Wulst *n* = 74; NCL *n* = 49) and done separately for every recorded region. For each neuron, each trial’s data was binned in 200 ms bins, with a sliding window of 40 ms. It was necessary to average the activity across trials of the same condition and temporally smooth the data, to minimize fluctuations originating in random spiking (Cunningham and Yu, 2014). Therefore, the data was averaged per condition (video × actor) and further smoothed with a 200 ms Gaussian kernel. Neural activity was also z-scored: for each neuron, the neuron’s average baseline spike count (calculated over a 2 s period, in the middle of the 6 s baseline) was subtracted to the averaged trial activity. The difference was subsequently divided by the standard deviation the baseline spike count. The last data processing step was to reduce the baseline to only 600 ms, for purposes of readability of the neural trajectories.

We build an activity matrix that concatenated the time resolved activity of all neurons, in each condition: to guarantee that we compare the same principal components across all conditions, the temporally resolved response of all neurons, to all combinations of video and actor, was considered pseudo-simultaneous (Ditz and Nieder, 2020; Ott and Nieder, 2016).

From the PCA, we obtained the three first principal components and calculated the trajectories for each condition. The assessment of the trajectories of each video allowed us to observe differences in population dynamics when the same video had pigeons or greebles on screen: these differences were quantified by the calculation of the Euclidean distances between the trajectories generated by the same video, when the actor was a pigeon or a greeble.

### Histology and electrode track reconstruction

At the end of the experiment the pigeons were euthanized with CO_2_ by placing them in a 450 mm (Length) × 340 mm (Width) × 300 mm (Depth) chamber, with a CO_2_ delivery rate of 16 L/min, an amount that equated to displacing 35% of the chamber volume per minute. The CO_2_ was left on for 5 min, and for at least 2 min beyond the point where the animal stopped breathing. The animal was then immediately perfused with 10% formalin in physiological saline. The brains were removed from the skull and kept in 10% formalin for at least 5 days, followed by sucrose formalin (10% formalin, 30% sucrose), and allowed to sink twice. The brains were frozen and sectioned at 40 μm sections with every 10th section mounted and stained with thionin.

## Results

### Electrode positions

All electrode tracks were within the borders of the targeted NCL, MVL, and Wulst regions as defined by Karten and Hodos (1967) and Kröner and Güntürkün (1999). For NCL, the intended track positions were AP +5.5 and ML ± 7.5. The track positions for two NCL birds (K6, X27) were as intended. The track position for K4 was AP +6.0, ML +7.0, differing from the intended AP and ML positions by 0.5 mm.

For MVL, the intended track positions were AP +10.5 and ML ± 6.0. The track positions for two MVL birds (JIM, PAM) were as intended. The position for K8 were AP +10.5, ML ± 5.5, differing only from the intended ML position by 0.5 mm.

**FIGURE 3:**
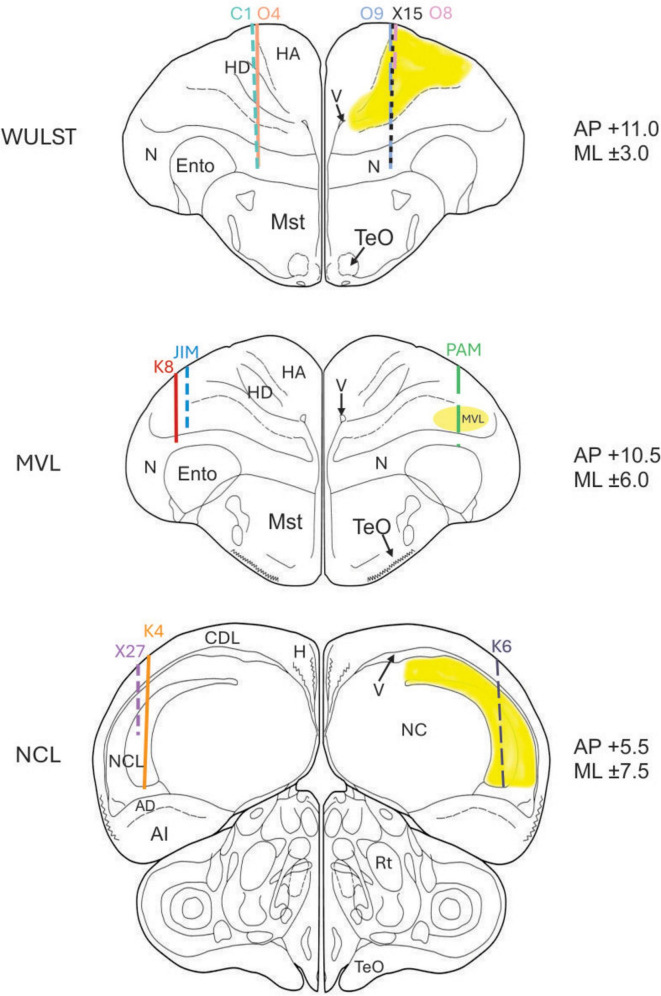
Electrode track records. Visualization of the electrode track reconstructions, for every pigeon (pigeon identifiers are shown on top of the respective track). The targeted brain regions are shaded in yellow, and the target coordinates are displayed to the right of the slices. Abbreviations: apical part of the hyperpallium (HA), densocellular part of the hyperpallium (HD), ventricle (V), nidopallium (N), mesopallium ventrolaterale (MVL), entopallium (Ento), medial striatum (MSt), tectum opticum (TeO), nucleus rotundus (Rt), hippocampus (H), dorsolateral corticoid area (CDL), nidopallium caudolaterale (NCL), dorsal arcopallium (AD), intermediate arcopallium (AI), caudal nidopallium (NC).

For Wulst the intended track positions were AP +11.0 and ML ± 3.0. The track positions for three Wulst birds (C1, O4, X15) were as intended. The position for the O8 and O9 were AP +10.75, ML ± 3.0, differing only from the intended AP positions by 0.25 mm (Figure 3).

### Single-unit analysis

In this experiment, we recorded neural activity in three regions of the pigeon brain (Wulst, MVL and NCL) during the passive visualization of videos depicting conspecifics or control shapes performing the following behaviors: courtship, eating, flying and walking. Half of the video presentations, of the pigeon videos as well as the control shapes, included the sound of the pigeon videos.

We recorded and analyzed 103 units in MVL, 123 in NCL and 213 units in Wulst. Data analysis began with the assessment of the data distribution of the recorded neuronal populations. For all regions, the negative binomial distribution had a better fit to the data (lower AIC) than the Poisson distribution (log likelihood ratio test between models for the MVL data: χ^2^(1) = 22042,*p* < 0.0001; NCL: χ^2^(1) < 22853,*p* < 0.0001; Wulst: χ^2^(1) = 9808.9,*p* < 0.0001). Furthermore, the negative binomial null models were not zero-inflated (MVL: ratio = 1.0067, *p* = 0.79; NCL: ratio = 1.0183, *p* = 0.38; Wulst: ratio = 1.0014, *p* = 0.88) and the residuals fitted the assumptions of the distribution.

We first assessed if the spike counts of each neuron were significantly modulated, compared to baseline, for the fixed effects of behavior (courtship, eating, flying and walking, and non-interactive two pigeons), actor (the video depicted a pigeon or a greeble) and the interaction between them. Additionally, we tested the interaction between the presence of sound and the actor on screen (GLMM per neuron: m1, described in Methods).

**FIGURE 4:**
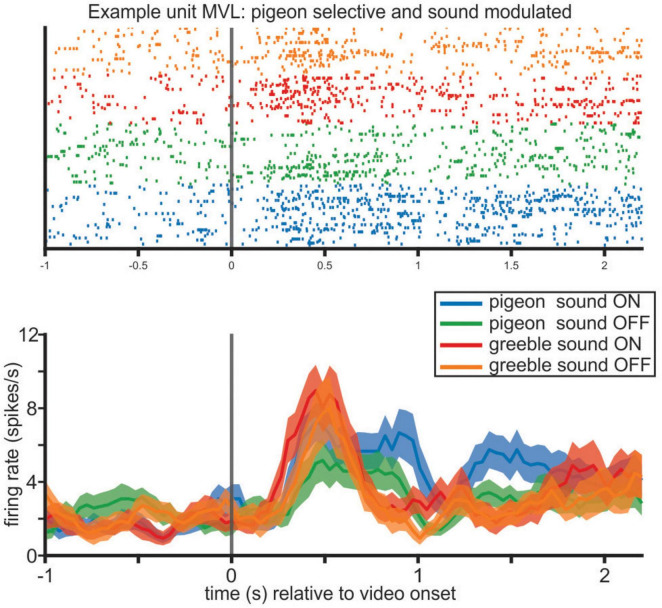
Example sound modulated unit in MVL. The output of the model (see Supplementary material) indicated that this neuron was pigeon selective, and sound modulated. The firing rate of the unit here depicted is differently modulated for the pigeon videos (blue and green traces) compared to the greeble videos (orange and red traces). There is a visible separation between presentations of the pigeon videos with sound compared to the presentations of the pigeon videos without sound.

In MVL, the activity of 65 out of 103 recorded neurons (63%) was task modulated. In this dataset, 15 units (23%) were categorically tuned to the presence of the actor: 7 (11%) units displayed the most amount of modulation when a pigeon was on screen; 5 (8%) were selective for the greebles, and 3 (5%) units had an excited response for the pigeon videos but an inhibited one for the greeble videos. Additionally, 2 of the units that coded for the pigeon were sound modulated: firing rates differed significantly between the trials in which the pigeon videos were displayed with sound on, compared to the same videos without sound, and the greeble videos with and without sound (Figure 4).

Moreover, 5 (8%) units were significantly modulated by one specific behavior, for both pigeons and greebles. 21 units (32%) were modulated by a specific behavior, performed only by one actor. We also found one unit that was selective for more than one behavior and 7 units that were predominately selective for the two pigeon controls. The remaining units were visually selective but not modulated by the content of the videos. Between the behaviorally modulated units and the exclusively visually selective units, 12 units presented a significant interaction between actor and sound and differentiated between the sound on and sound off presentations of the pigeon or greeble videos.

We applied the same categorical analysis (model m1) to the 213 units recorded in Wulst. We found 91 task modulated units. The activity observed was best explained by the presence of a pigeon on screen in 13 units (14%). Other 11 units (12%) fulfilled the criteria of having a significant interaction between the greeble and at least 2 behaviors or exclusively having a significant main effect for the actor greeble. 9 units (10%) were classified as behavior selective. Furthermore, 28 units (31%) were selective for a specific behavior performed only by one actor. We also found 7 (8%) units predominantly modulated by the two-pigeon stimulus set and 3 (3%) that responded to two behaviors. The remaining units had a generalized visual response to all stimulus categories. In this region, there were also 4 (4%) sound modulated units, with one of them being selective for the actor pigeon.

Lastly, we recorded 123 units in NCL. 60 (49%) neurons were task modulated. This dataset had 5 (8%) units in which the presence of a pigeon on screen drove the most amount of neural modulation, and other 5 (8%) units that were modulated by the greebles. We also found 9 (15%) units that encoded one specific behavior for both actors. There were also 3 (5%) units that encoded two behaviors and 3 (5%) other units that encoded two behaviors, each one performed by a different actor. 15 (25%) units encoded a specific behavior performed only by one actor. The remaining units have a significant main effect for both the pigeon and the greeble and no further differentiation amongst the behavioral conditions. That is, they are modulated by the presence of a stimulus in a way that is not specific to the content of the video. They were interpreted as visually selective. Regarding sound modulation, we found 7 (12%) units that were sound modulated, but none belonged to the ones labeled as pigeon selective.

**FIGURE 5:**
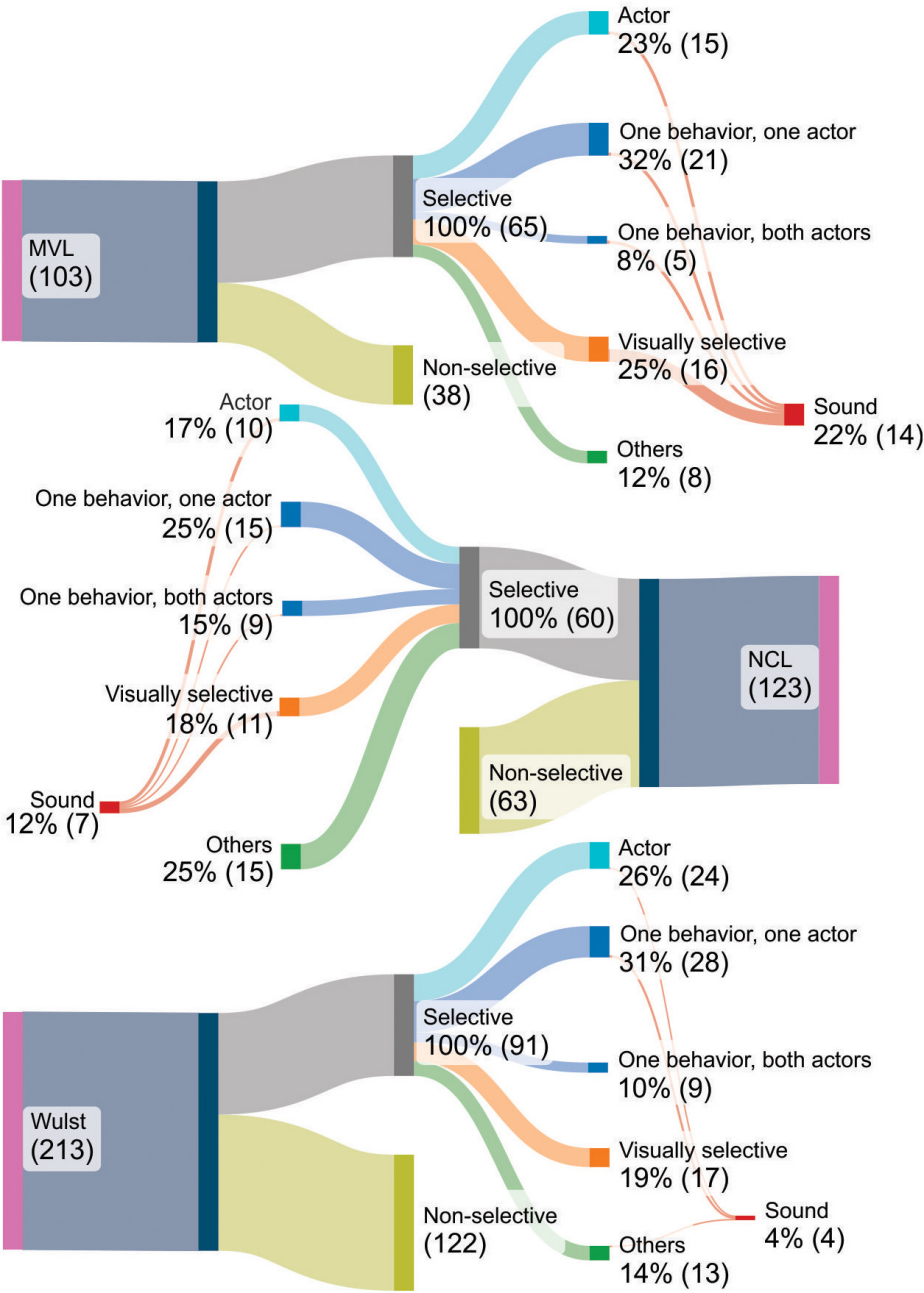
Number of units with a significant response to the categorical task variables for every region, displayed as percentage (rounded) and absolute counts After fitting each unit’s activity with the m1 model, neurons were classified as actor-selective (if exclusively selective for one actor, during at least two behaviors), behavior-selective (if exclusively selective for one behavior, performed by both actors—labeled “one behavior, both actors” in this figure) or actor-and-behavior-selective (if were only modulated by one actor performing one behavior–labeled “one behavior, one actor” in this figure). Units that were modulated by the presence of a stimulus without any further distinction were labeled visually selective; the ones not task modulated were labeled non-selective; units selective for more than one behavior or for the two-pigeon stimulus set are included under the category “others.” Lastly, sound modulated neurons were also accounted for.

The proportion of units (Figure 5) classified as actor selective did not differ between the three recorded regions [χ^2^(2) = 2.34, *p* = 0.3], and the same was the case for the proportion of units classified as behavior selective [χ^2^(2) = 1.54, *p* = 0.46]. However, there were differences regarding the proportion of sound modulated units: MVL had a higher proportion of units classified as sound modulated compared to Wulst [χ^2^(1) = 17.7, *adjustedp*-*value* = 0.0001]. No significant differences were found between NCL and MVL [χ^2^(1) = 4.15, *adjustedp*-*value* = 0.08] or, NCL and Wulst (*Fisher*′*sExacttest*:*adjustedp*-*value* = 0.11).

### Population analysis

Although only a small percentage of neurons were considered pigeon selective, we were interested in assessing which moments in the video drove the most amount of modulation. We fitted the subpopulations of pigeon-responsive neurons, grouped by region, with model *m2* (see Methods). For every video, the 200 ms bins in which the populational activity significantly differed from the populational baseline were identified. Subsequently, in each of these bins, the activity recorded during the presentation of a pigeon video and the respective greeble control was compared.

**FIGURE 6:**
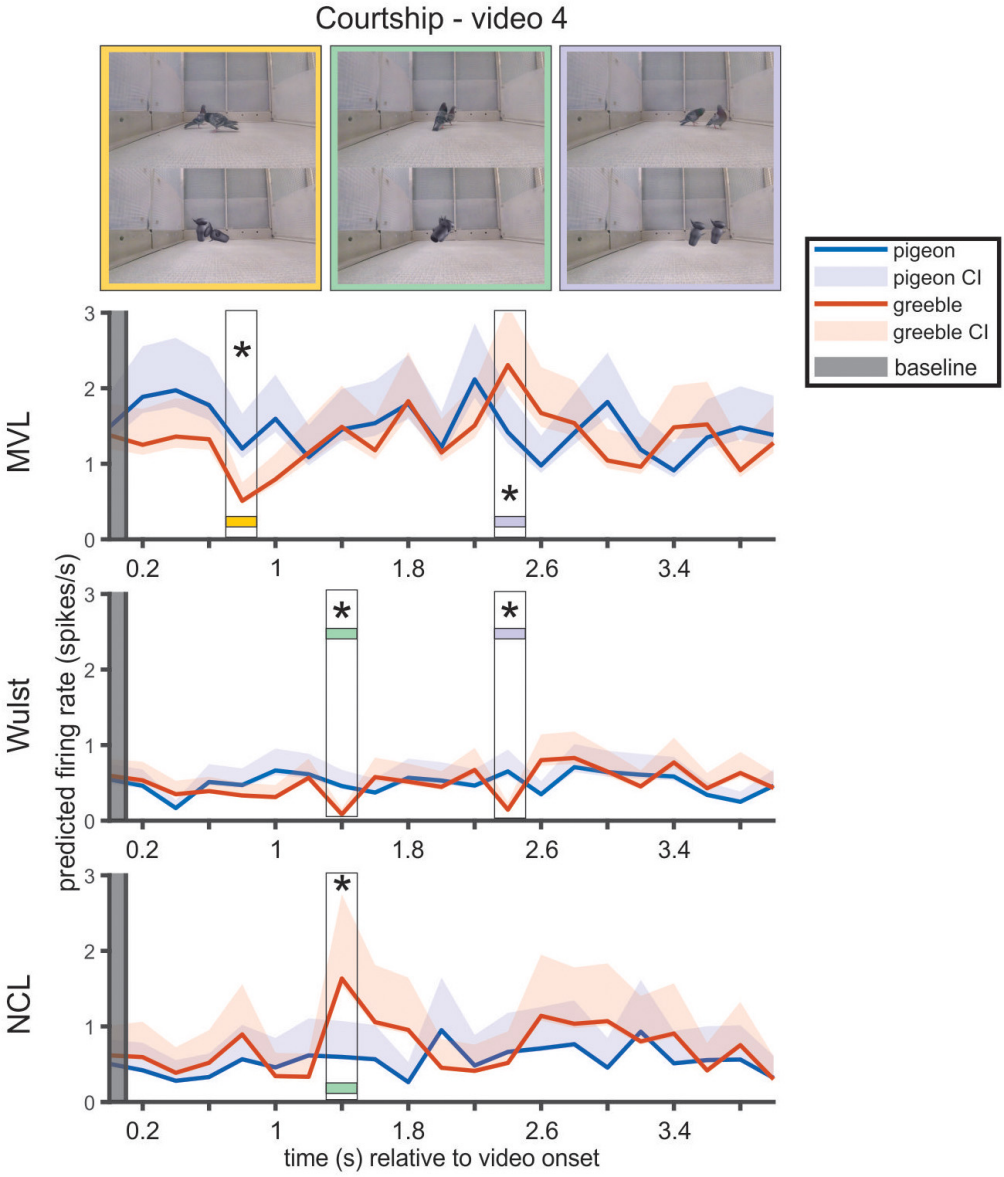
Time points in courtship video 4 that modulated the subpopulation of pigeon responsive neurons. For each region, the neurons labeled as pigeon selective were grouped and fitted with the model m2. We obtained the estimated marginal means for the interaction between every time bin in the video and each actor. Hence, the panels for MVL, Wulst and NCL display the population’s response predicted by the model. The time points in which the subpopulation’s activity significantly differs from the subpopulation’s baseline are highlighted with a box. The asterisk indicates a significant difference in subpopulation response between the pigeon and greeble presentations of the video. Video frames displayed during the highlighted time bins are presented at the top of the figure and matched to the respective time bin with a colored band.

In Wulst and MVL, the populations diverged from their respective baselines, for at least a period of 200 ms, in most videos (MVL: 11 out of 16; Wulst: 12 out of 16). In the videos depicting courtship or eating behaviors, every time bin that significantly differed from baseline corresponded to frames where the animal was performing a bowing motion or walking (Figure 6). During flying videos, the significant moments depicted wing flapping and, in one instance for both MVL and Wulst, small body movements while the animal was stationary. In walking videos, all relevant bins corresponded to the animal moving forward (relative to its original trajectory).

In MVL, 2 out of 11 videos had bins where there was a significant difference of activity between the actor pigeon, and the actor greeble. Both instances depicted the animal walking. In Wulst, 9 out of 12 videos contained bins where activity significantly differed from baseline and differed between the pigeon and the greeble presentations of the video.

In NCL, only 5 out of 16 videos had bins where the population activity was significantly modulated from the populational baseline. Furthermore, the identified bins did not differ between the pigeon and the greeble presentations.

**FIGURE 7:**
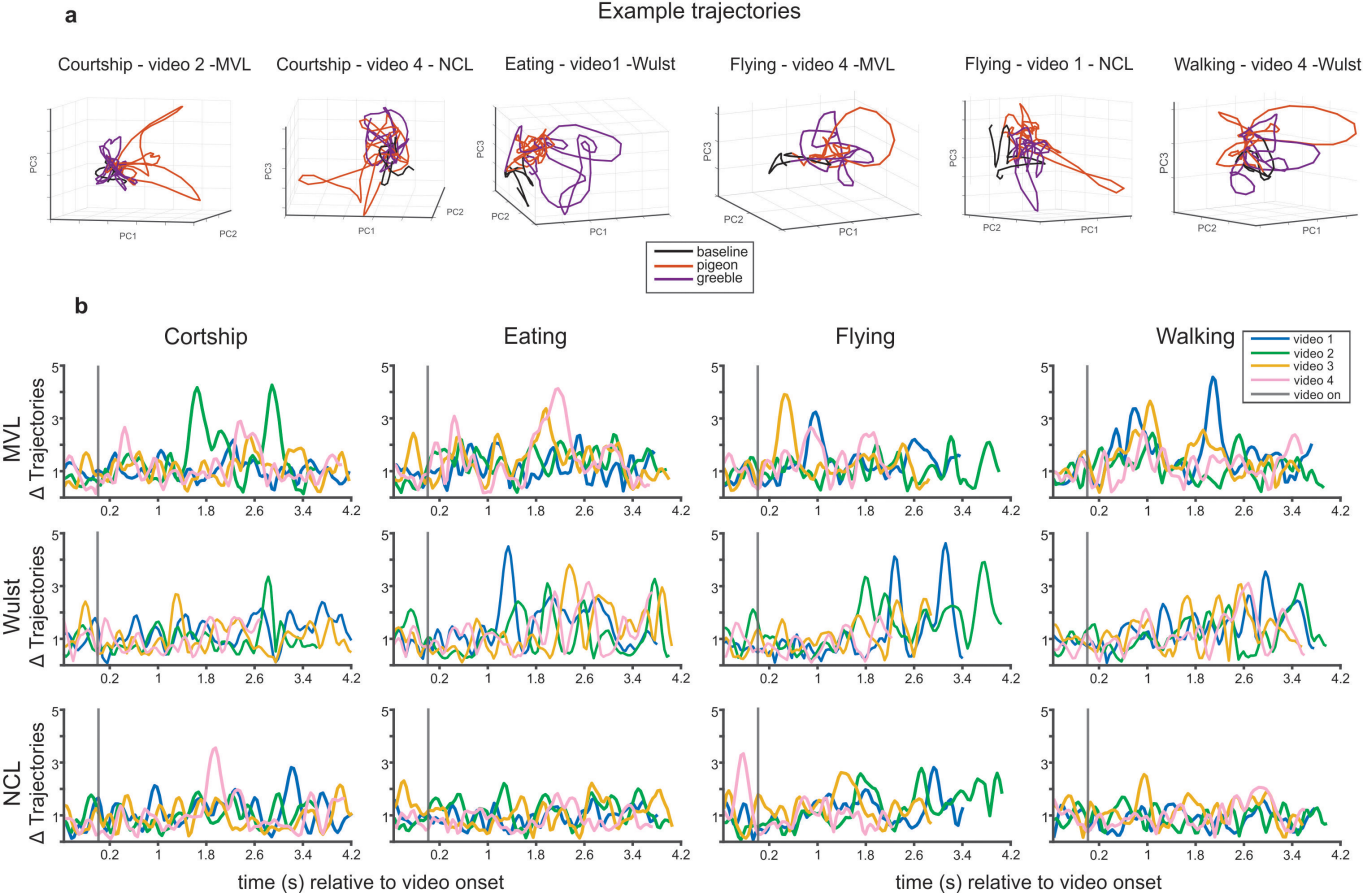
State-space analysis of population dynamics during the presentation of each video. **(a)** State-space trajectories for 6 videos. The trajectories were plotted separately for the actor pigeon and actor greeble presentations of the same video. There are brief moments throughout the presentation of videos in which the population represents the actors differently. **(b)** Euclidean distances between the actor pigeon and actor greeble presentations of the same video.

Lastly, we analyzed the populational dynamics of the representations, generated by each stimulus, with a population state-space analysis. The aim of this analysis was to identify patterns of activity that differ between the representation of the pigeons and greebles (see Methods for details). The trajectories (Figure 7a) describe instantaneous activation patterns, learned from the data (Pang et al., 2016): they represent the progression of the populational response, during 600 ms of baseline and the entire video presentation, for each condition (video x actor). The trajectories were projections of the first three principal components on the data. The first three principal components contain 17% of the populational variance in MVL, 13% in Wulst and 11% in NCL (Supplementary Figure 2).

In MVL and Wulst, it is possible to observe a destabilization of the population baseline activity, for every behavioral category (Figure 7). Within each category, the videos differ regarding how much separation there is between the trajectories of the actor pigeon and the actor greeble. We quantified the differences in trajectories by measuring the Euclidean distance between them, at every timepoint (Figure 7b). The moments in which the neuronal populations distinguish between the pigeons and the greebles occur in brief periods, not in a sustained way: the distances between trajectories return to values like the ones observed during the baseline after a visible peak.

For each video, we identified the two time points with the largest Euclidian distance between trajectories. During courtship videos, the biggest differences between activity related to pigeons and greebles were found while the pigeon performed bowing motions and walking. During eating videos, it coincided with bowing motions, pecking, mandibulation and walking. In the flying videos, the differences corresponded to two distinct types of behavior. On one hand, moments where the pigeon was flapping its wings and, therefore, creating a lot of movement on screen, but also in moments where the animal was stationary and only performing slight body shifts, such as small head movements. Lastly, in the walking videos, the differences between pigeons and greebles corresponded to the coordinated motion of stepping and head-bobbing. All behaviors were represented in the modulation of the population of every region.

## Discussion

We recorded single-unit responses in three different regions of the pigeon brain (MVL, Wulst, and NCL) while head-fixed birds watched videos of conspecifics and control shapes performing four different behaviors: courtship, eating, flying, and walking. We aimed to assess if there is specific neuronal coding for conspecifics at different structures of the pigeon’s visual system when exposed to dynamic, naturalistic stimuli.

In each region, we found a small proportion of units that were modulated by the pigeon videos. However, we did not find a difference between the regions regarding the proportions of units that responded to the actors or the behaviors observable in the videos. The response patterns we observed were neither in line with primate face-cell coding in terms of selectivity to a particular body feature like the head (Desimone et al., 1984), nor did they display an invariant, abstract response to social content (McMahon et al., 2015). This lack of a simple, categorical “conspecific” code suggests a different processing strategy in the pigeon brain. Our findings are consistent with a tuning landscape model (Ponce et al., 2019; Wang and Ponce, 2022) of visual processing, like that described in the primate ventral stream. In this framework, neurons in the higher centers of the pigeon visual system are not simple category detectors, but are better viewed as signaling distances to specific, preferred combinations of visual attributes within a high-dimensional feature space.

Our population analysis supports our interpretation. We found that population activity in the visual areas (MVL and Wulst) showed modulations in discrete moments of the video, corresponding to specific motions like bowing, wing flapping, and head-bobbing. We interpret these momentary differences as the points where the pigeon video trajectory and the greeble video trajectory diverged on this shared, high-dimensional landscape. The greeble, by not emulating the species-specific motion kinematics, may have simply traced a different path through this feature space, resulting in a momentarily separable neural state. We believe that the distinctions in encoding that we observed was not a categorical social signal but rather a discrepancy of visual features over time (see Supplementary Figure 1). To verify this possibility, future work should model the kinematics of the videos specifically (e.g., head angular velocity, body displacement) to look for modulation of population activity that aligns with changes in these kinematic variables during the videos.

The absence of a clear difference in the NCL subpopulation reinforces our view that visual areas employ sparse coding of visual stimuli. NCL, the avian prefrontal equivalent, is known to encode task-relevant visual information for active categorization (Divac et al., 1985; Johnston et al., 2017). Its lack of any differential engagement during this passive task—while visual areas (MVL/Wulst) showed feature-driven modulation—is consistent with a functional division between visual feature processing and higher-order cognitive engagement in NCL. The present experiment did not require an explicit categorization of the stimuli during passive fixation, as is often the case in operant chamber tasks (Scarf et al., 2016; Clark et al., 2019; Clark et al., 2022a,b; Dittrich et al., 1998).

A separate finding was the presence of sound-modulated visual units, with a greater proportion in MVL compared to Wulst. This points to the tectofugal pathway (which includes MVL) as a site for audio-visual integration, building on earlier reports of auditory responses in the pigeon optic tectum (Lewald and Dörrscheidt, 1998). This integration may be important for behavior, as other studies have shown that pigeons use vocalizations to aid in visual categorization (Partan et al., 2005). While the proportion of such neurons in our dataset was small, their presence in MVL suggests multimodal integration may occur early in the avian hierarchy of sensory processing. Sound modulated neurons that are primarily visually responsive are, in fact, found in the primate anterior fundus face patch (Khandhadia et al., 2021). These face selective neurons display enhanced responses to faces of conspecifics when the stimuli were accompanied by the conspecific’s vocalization. In the present experiment, although we find neurons that modulate their response to videos of pigeons with sound, their proportion was quite low as part of the population we sampled, making it difficult to know if these represent a genuine feature of the MVL.

A known problem of experiments that use videos to study the processing of social stimuli in pigeons is the color display (Ware et al., 2015). Pigeons have tetrachromatic color vision, making it likely that colored videos of conspecifics, displayed on monitors optimized for the human visual system, do not look very realistic. Nonetheless, studies that assessed pigeons’ behavioral responses towards videos of conspecifics, displayed without the UV component, report that animals maintain natural responses towards them (Shimizu, 1998; Ware et al., 2015; Ware et al., 2017). Therefore, pigeons are still able to extract relevant social information from video stimuli, displayed with suboptimal colors. At the neuronal level, sensitivity to colors in the UV range has been reported in Wulst (Nimpf et al., 2024). We expect videos of social scenes with naturalistic color information to be represented in MVL and Wulst similarly to the ones used in the present experiment, where video features are encoded at the population level. Nonetheless, we would expect a higher response range from the individual neurons in these regions, evoked by more naturalistic color information, that reflect the distance to the neuron’s preferred “prototype.” Taken together, the findings of the experiment suggest that the pigeon visual system, much like the primate visual cortex, does not employ a simple, invariant code for “conspecifics.” Instead, our findings support a high-dimensional tuning landscape model, where populations in MVL and Wulst encode specific features that change over time in dynamic videos.

## Data Availability

The raw data supporting the conclusions of this article will be made available by the authors, without undue reservation.
