# Supplementary material for: Single neuron responses in NCL, MVL, and Wulst during the observation of videos of conspecifics support population feature coding
Source: Front Behav Neurosci. 2026 Feb 26;20:1736261. doi: 10.3389/fnbeh.2026.1736261 (PMC12979531; doi:10.3389/fnbeh.2026.1736261)
Supplement: Supplementary file 1 [file Data_Sheet_1.docx]

# Supplementary material

## Supplementary material to the processing of stimuli

To quantify how visually dissimilar each video was from its control, we performed a frame-by-frame correlation. Considering that the stimulus set is in color, we started by separating the three RGB layers of each frame. Then, a correlation between every color layer of the pigeon video’s frame and the corresponding color layer of the greeble video’s frame was performed. The obtained values were smoothed with a 200ms gaussian kernel (for ease of comparison with the results of the population state-space analysis – Results, figure 7). We also provide the correlation values obtained when the images are converted to grayscale (figure S1).

The correlation between frames was relevant for the interpretation of the population state-space analysis: we interpreted the observed modulation in population activity, in the visual areas (MVL and Wulst), as moments where the pigeon video trajectory and the greeble video trajectory diverge in a high-dimensional feature space. The moments of divergence correspond to species-specific motion, displayed differently by the pigeon videos and the controls. It was important to explore an alternative interpretation: whether the modulations observed can be explained by a high degree of dissimilarity between images, instead. If this was the case, we would expect to observe decreases in correlation between frames, where we also observe the highest difference between the trajectories of population activity. However, the correlation between images stays stable, in moments where the neuronal population activity differs between the processing of the pigeon and the greeble version of a video (e.g. figure S1 – courtship and eating videos). Drops in correlation between frames do not seem to influence the divergence in population activity (e.g. figure S1 – video Flying 3).

**
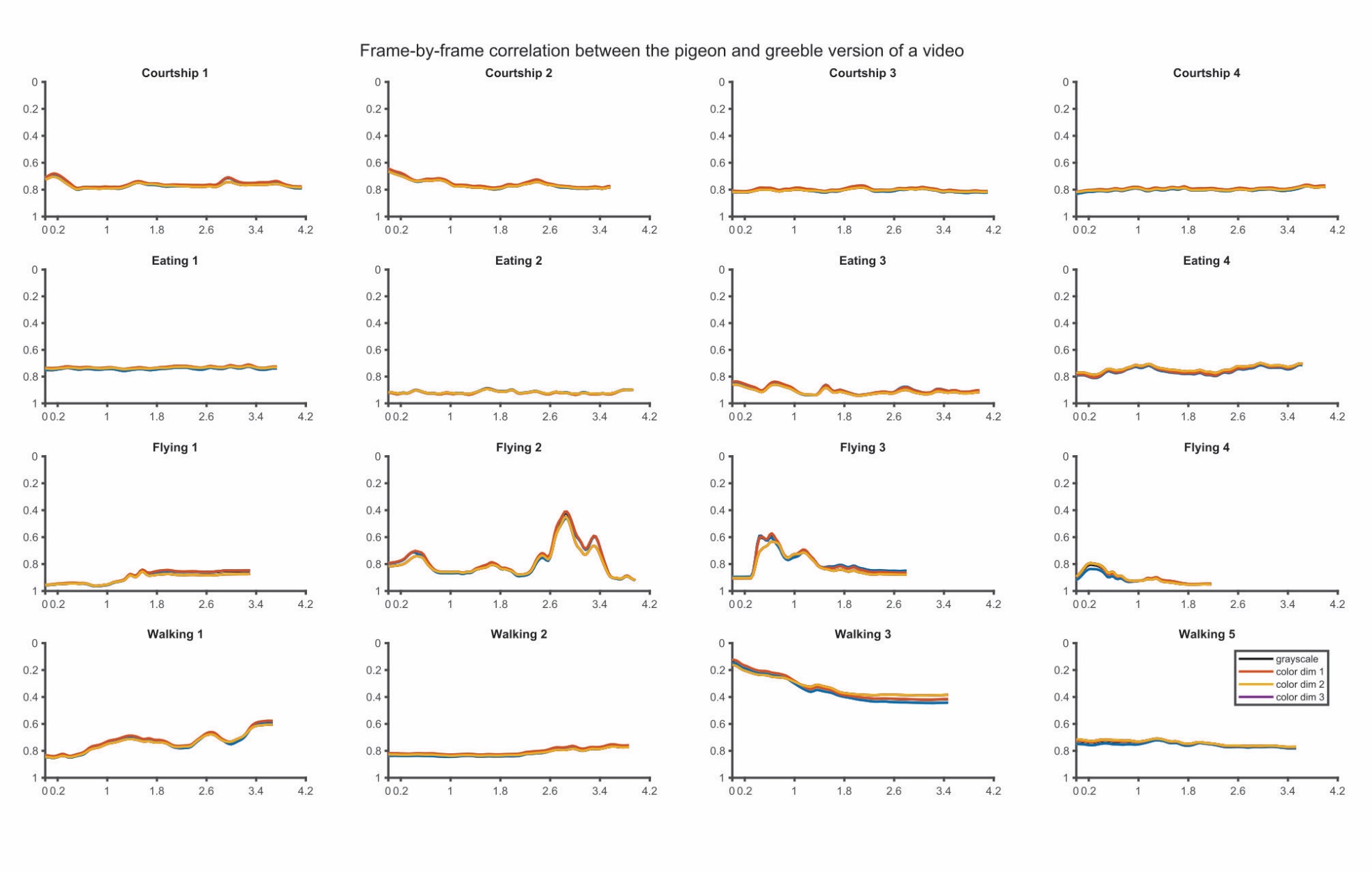
Figure S1:** Frame-by-frame correlation between each pigeon video and its respective control. Oscillations in correlation values were first assessed for each RGB color layer, of every frame (here plotted as “color dim 1”, “color dim 2” and “color dim 3”). We also looked at the oscillation in correlation values when the images were converted to grayscale (here plotted as “grayscale”). This analysis was relevant for the interpretation of the trajectory differences between a pigeon video and its control. We were interested in observing if decreases in frame correlation occurred where we also observe the highest difference between the trajectories of population activity. To ease the comparison between figure S1 and figure 7b, the y-axis of each plot was inverted.

##
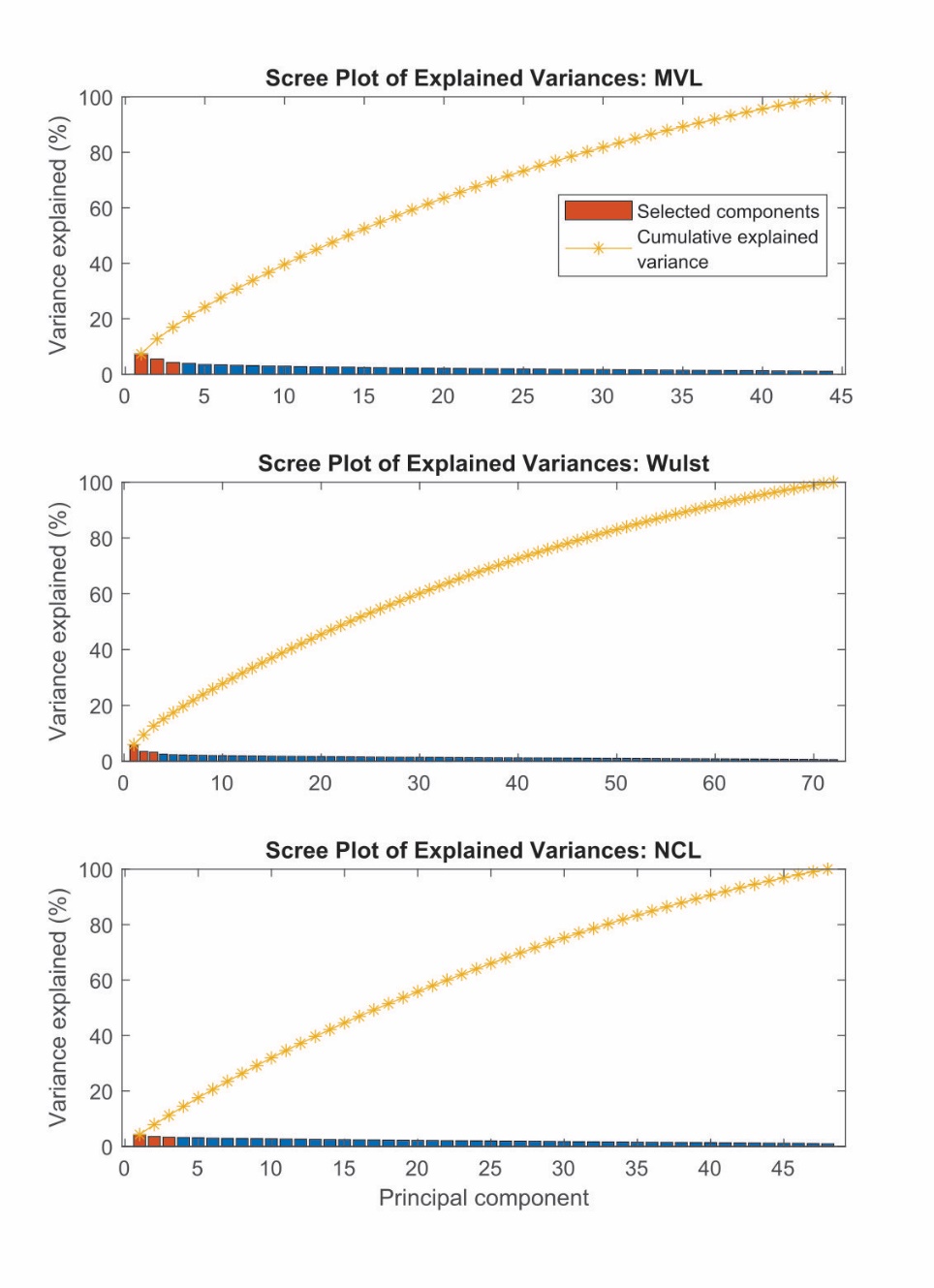
Supplementary material to the population state-space analysis

**Figure S2**: Scree plot of explained variances of the three recorded regions. For every region, a principal components analysis was performed on a matrix that concatenated the time resolved activity of all neurons (averaged within experimental conditions), across all conditions (video x actor). The first three principal components contain 17% of the populational variance in MVL, 13% in Wulst and 11% in NCL. The first three principal components were selected for the evaluation of population dynamics.

## Supplementary material to single-unit results - example neurons

**Figure 4 example neuron**

|  | **Figure 4 example neuron** | | | |
| --- | --- | --- | --- | --- |
| *Predictors* | *Log-Mean* | *CI* | *Statistic* | *p* |
| Intercept | -0.82 | -1.03 – -0.61 | -7.57 | **<0.001** |
| Actor (pigeon) | 0.12 | -0.15 – 0.39 | 0.89 | 0.373 |
| Actor (greeble) | 0.08 | -0.19 – 0.36 | 0.59 | 0.556 |
| Behavior (eating) | 0.10 | -0.17 – 0.36 | 0.73 | 0.466 |
| Behavior (flying) | -0.05 | -0.32 – 0.22 | -0.37 | 0.714 |
| Behavior (walking) | -0.10 | -0.37 – 0.18 | -0.69 | 0.487 |
| Behavior (2pigeonContol) | 0.15 | -0.17 – 0.47 | 0.92 | 0.357 |
| Sound (on) | -0.10 | -0.28 – 0.08 | -1.11 | 0.268 |
| Actor (pigeon) x Behavior (eating) | 0.10 | -0.24 – 0.43 | 0.56 | 0.573 |
| Actor (greeble) x Behavior (eating) | 0.31 | -0.03 – 0.64 | 1.77 | 0.078 |
| Actor (pigeon) x Behavior (flying) | 0.23 | -0.12 – 0.58 | 1.28 | 0.200 |
| Actor (greeble) x Behavior (flying) | 0.35 | -0.00 – 0.71 | 1.94 | 0.053 |
| Actor (pigeon) x Behavior (walking) | 0.30 | -0.05 – 0.64 | 1.69 | 0.091 |
| Actor (greeble) x Behavior (walking) | 0.20 | -0.16 – 0.55 | 1.09 | 0.275 |
| Actor (pigeon) x Behavior (2pigeonContol) | 0.01 | -0.36 – 0.38 | 0.04 | 0.971 |
| Actor (pigeon) x Sound (on) | 0.41 | 0.19 – 0.63 | 3.63 | **<0.001** |
| Actor (greeble) x Sound (on) | 0.19 | -0.05 – 0.43 | 1.52 | 0.128 |
| **Random Effects** | | | | |
| σ^2^ | 1.26 | | | |
| τ_00_ _trialNr_ | 0.08 | | | |
| ICC | 0.06 | | | |
| N _trialNr_ | 216 | | | |
| Observations | 6091 | | | |

**Table 1:** Result of a generalized linear mixed model (GLMM) applied to the data of the example unit shown in Figure 4. Firing rate significantly increases, relative to baseline, when the pigeon videos are displayed with sound on. The intercept of this model is the spike counts during a period of two seconds, in the middle of the baseline, with no actor present and no sound. Model: spikeCounts ~ actor*behavioralCategory+actor*sound +(1 | trialNumber)
